# Amyloid plaque formation precedes dendritic spine loss

**DOI:** 10.1007/s00401-012-1047-8

**Published:** 2012-09-21

**Authors:** Tobias Bittner, Steffen Burgold, Mario M. Dorostkar, Martin Fuhrmann, Bettina M. Wegenast-Braun, Boris Schmidt, Hans Kretzschmar, Jochen Herms

**Affiliations:** 1 Center for Neuropathology, Ludwig-Maximilians University, Feodor-Lynen-Strasse 23, 81377 Munich, Germany; 2 Department of Translational Brain Research, DZNE-German Center for Neurodegenerative Diseases, Munich, Ludwig-Maximilians-University, Feodor-Lynen-Str. 23, 81377 Munich, Germany; 3 DZNE-German Center for Neurodegenerative Diseases, Bonn, Sigmund-Freud-Strasse 25, 53105 Bonn, Germany; 4 Department of Cellular Neurology, Hertie Institute for Clinical Brain Research, University of Tübingen, Ottfried-Müller-Strasse 27, 72076 Tübingen, Germany; 5 DZNE-German Center for Neurodegenerative Diseases, Tübingen, Tübingen, Germany; 6 Clemens Schoepf-Institute for Organic Chemistry and Biochemistry, Technische Universität Darmstadt, Petersenstrasse 22, 64287 Darmstadt, Germany

**Keywords:** Alzheimer’s disease, Two-photon in vivo imaging, Dendritic spines, Amyloid plaque, Kinetics, Structural plasticity

## Abstract

Amyloid-beta plaque deposition represents a major neuropathological hallmark of Alzheimer’s disease. While numerous studies have described dendritic spine loss in proximity to plaques, much less is known about the kinetics of these processes. In particular, the question as to whether synapse loss precedes or follows plaque formation remains unanswered. To address this question, and to learn more about the underlying kinetics, we simultaneously imaged amyloid plaque deposition and dendritic spine loss by applying two-photon in vivo microscopy through a cranial window in double transgenic APPPS1 mice. As a result, we first observed that the rate of dendritic spine loss in proximity to plaques is the same in both young and aged animals. However, plaque size only increased significantly in the young cohort, indicating that spine loss persists even many months after initial plaque appearance. Tracking the fate of individual spines revealed that net spine loss is caused by increased spine elimination, with the rate of spine formation remaining constant. Imaging of dendritic spines before and during plaque formation demonstrated that spine loss around plaques commences at least 4 weeks after initial plaque formation. In conclusion, spine loss occurs, shortly but with a significant time delay, after the birth of new plaques, and persists in the vicinity of amyloid plaques over many months. These findings hence give further hope to the possibility that there is a therapeutic window between initial amyloid plaque deposition and the onset of structural damage at spines.

**Electronic supplementary material:**

The online version of this article (doi:10.1007/s00401-012-1047-8) contains supplementary material, which is available to authorized users.

## Introduction

Alzheimer’s disease (AD) is the most common form of dementia, which is characterized by two neuropathological hallmarks, namely β-amyloid plaque deposition and intracellular neurofibrillary tangles. The progression of disease pathology is further accompanied by a substantial loss of neurons and synapses. Controversial findings have, however, been reported regarding the relationship of synapse loss and plaque load [3, 33]. For instance, synapse loss has been reported as an early event in the brains of AD patients and it represents the pathological feature that best correlates with cognitive impairment [11, 23, 26, 39, 47, 48, 56]. Indeed, AD pathology includes a substantial decrease in the number of dendritic spines [16, 33, 44], which are thought to be a structural correlate of learning and memory [2, 43, 62]. On the other hand, a strong correlation between cognitive impairment in AD patients and amyloid plaque load has also been described [42]. Amyloid plaques are associated with several pathological changes within and around them. For instance, plaques are associated with substantial inflammatory responses including activation of microglia and astrocytes [13, 38, 57]. Furthermore, neuritic changes and dystrophies are observed, which are accompanied by dendritic spine loss in the peri-plaque region [19, 60]. Within neuritic plaques, presynapses are almost completely lost [36]. These pathological features of amyloid plaques, observed in human beings, are mimicked in cortical and hippocampal brain areas of some AD mouse models [14]. Dendritic spine loss has been extensively investigated in the absence of plaques [1, 6, 28, 35, 45] as well as in their vicinity, in various AD mouse models [6, 19, 32, 34, 40, 53, 54, 59]. However, little is known about the underlying kinetics of these processes. For instance, Tsai et al. [59] reported an increased spine elimination and formation over 4–5 weeks, whereas Spires-Jones et al. found only increased spine elimination over 1 h [37, 53]. To shed more light on the chronology of these events, we investigated dendritic spines over several weeks in a well-characterized AD mouse model [7, 22, 46]. We focused on two age cohorts: (1) 3–4 months of age, when amyloid pathology is still dynamically unfolding, and (2) 18–19 months, which represents the phase of late-stage pathology. The kinetics of dendritic spine formation and elimination were analyzed in the vicinity and further away from plaques in AD transgenic mice as well as in control animals within somatosensory cortex. At the same time, we monitored the plaque growth kinetics. This combined approach enabled, for the first time to the best of our knowledge, to image individual dendritic spines before and during the appearance of amyloid plaques and hence correlate their respective kinetics. A similar approach has recently been applied to investigate the sequence of neuritic and glial changes [41, 52].

## Materials and methods

### Transgenic mice

APPPS1 mice are double transgenic for APP_KM670/671NL_ and PS1_L166P_ mutations [46]. We want to thank Matthias Jucker from the University of Tübingen and German Center for Neurodegenerative Diseases (DZNE), Tübingen, Germany, who kindly provided these mice. Heterozygous mice of this line were crossed with mice heterozygous for YFP-H [15] (B6.Cg-Tg(Thy1-YFPH)2Jrs/J from The Jackson Laboratory, Bar Harbor, USA). Heterozygous triple transgenic offspring of mixed gender were used in the experiments. Single transgenic YFP-H littermates were used as controls. Mice were group-housed under pathogen-free conditions until surgery, after which they were singly housed. All procedures were in accordance with an animal protocol approved by the University of Munich and the government of Upper Bavaria (Az. 55.2-1.54-2531-110-06).

### Cranial window surgery

A cranial window over the right cortical hemisphere was surgically implanted as previously described [5, 17]. The mice were anesthetized with an intraperitoneal injection of ketamine/xylazine (0.13/0.01 mg g^−1^ body weight). Additionally, dexamethasone (0.02 ml at 4 mg ml^−1^) was intraperitoneally administered immediately before surgery [25]. A circular piece of the skull over the somatosensory cortex (4 mm in diameter) was removed using a dental drill (Schick-Technikmaster C1; Pluradent; Offenbach, Germany). This was immediately covered with a circular glass coverslip (5 mm in diameter), which was glued to the skull using dental acrylic (Cyano-Veneer fast; Heinrich Schein Dental Depot, Munich, Germany) to close the craniotomy. A small metal bar, containing a hole for a screw, was glued next to the coverslip to allow repositioning of the mouse during subsequent imaging sessions. After surgery, mice received a subcutaneous analgesic dose of carprophen (Rimadyl; Pfizer, New York, NY, USA) for 3 days (5 mg kg^−1^). Imaging began following a 21-day recovery period after surgery.

### Long-term two-photon in vivo imaging

Long-term two-photon in vivo imaging was performed as previously described [5, 18, 29]. Less than 50 mW of laser power was delivered to the tissue to avoid laser-induced phototoxicity. For amyloid plaque staining, methoxy-X04 [31] (1 mg/kg) was intraperitoneally injected 24 h before imaging. YFP and methoxy-X04 were excited by a Ti:Sa laser (MaiTai, Spectra-Physics, Darmstadt, Germany) at 880 and 750 nm and the emission was collected from 527 to 582 nm and 460 to 500 nm, respectively (LSM 7 MP, Zeiss, Jena, Germany). For overview images, *z*-stacks of 230 × 230 × 150 μm^3^ with 2 μm *z*-resolution and 1024 × 1024 pixels per image frame (0.22 μm/pixel) were taken with a 40× IR-Achroplan water immersion objective (0.8 NA, Zeiss, Germany) to analyze amyloid plaques. For higher-resolution images to count dendritic spines, the same objective was used with 1 μm *z*-resolution and 512 × 512 pixels per image frame (0.11 μm/pixel). The high-resolution (2048 × 2048 pixels with 0.14 μm/pixel) and large volume (283 × 283 × 300 μm^3^) images with 2 μm *z*-resolution to monitor dendritic spines before and after plaque deposition were taken with a 20× W Plan-Apochromat water immersion objective (1.0 NA, Zeiss, Germany).

### Image processing and data analysis

All images were deconvolved using the adaptive blind 3D deconvolution algorithm of AutoDeblur with ten iterations (Version x2.0.1, Media Cybernetics Inc., Bethesda, MD, USA). The images were maximum intensity projected (Imaris 6.1, Bitplane, Zurich, Switzerland). In some figures, distracting neighboring dendritic elements were removed. Spines were counted in *z*-stacks by manually scrolling through the images of subsequent time points of the same position. The spine scoring method has previously been described [5, 18, 25]. Spine densities refer to the amount of spines per dendrite length in μm from which they protrude. Spine densities investigated on dendrites closer than 50 μm from a plaque were only analyzed on the segment that is located within the 50 μm radius from the plaque border. Spine morphology classification was performed by using the filament tracer module for 3D reconstruction in Imaris software (Version 7.4.2, Bitplane, Zurich, Switzerland). The volume measurements were done with the following parameters: small diameter 0.112 μm, large diameter 2 μm, and contrast threshold 0.3. Dendritic spines were classified due to their morphology into thin, stubby, and mushroom spines [21]. Filopodia spines were not found in the investigated brain region at 18–19 months of age. The geometrical classification rules from Harris et al. were interpreted by the following hierarchical expressions in the Imaris XT spine classification module: mushroom spines = “max_width(head)/min_width(neck) >1.4 and max_width(head) >0.5 and min_width(neck) >0”; stubby spines = “length(spine)/mean_width(neck) ≤3 or min_width(neck) = 0 or min_width(neck) >0.5”; thin spines = “length(spine)/mean_width(neck) >3” [29]. The volume of amyloid plaques was automatically calculated using Imaris software (Version 6.2, Bitplane, Zurich, Switzerland) as previously described [8]. The size of new born plaques was directly presented as volume (Fig. 5), whereas for pre-existing plaques that were already present at the first imaging time point the radius was calculated assuming a spherical plaque shape [22]. All data are presented as mean ± SD or ±SEM or 95 % confidence intervals (95 % CI). Error types are stated where appropriate. Statistical differences in measurements over time were determined using repeated-measures ANOVA while statistical comparison between two groups was performed with Student’s *t* test. Multiple group comparison was done by one-way ANOVA followed by Tukey–Kramer post hoc test. The slope from a linear regression was tested for statistical difference from zero by *F* test. Distributions of morphological subtypes of dendritic spines were tested for differences by Chi-square test. All statistical analysis and graphs were done using Prism 5.04 (GraphPad Software Inc., La Jolla, CA, USA). Figures were arranged using Adobe Illustrator CS4.

## Results

**Fig. 1:**
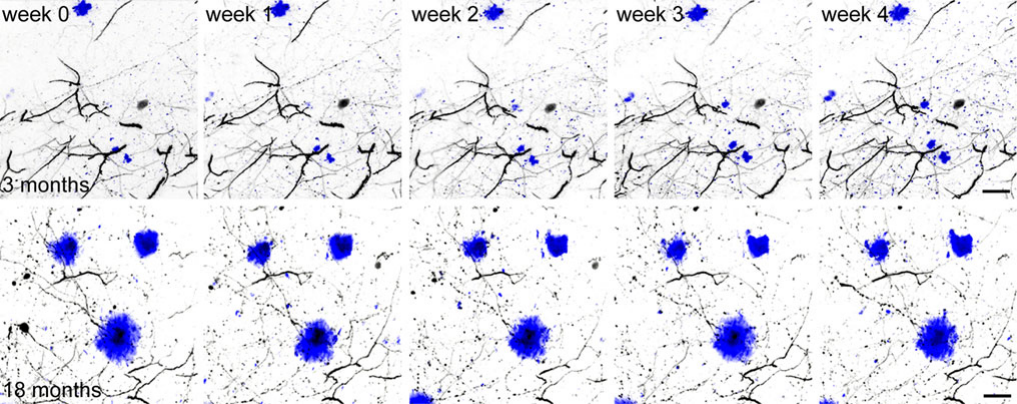
Time series of two-photon in vivo overview fluorescence images showing methoxy-X04 labeled amyloid plaques in *blue* and YFP-labeled dendrites in *grey* from mice 3–4 months and 18–19 months of age. *Scale bar* represents 20 μm

**Fig. 2:**
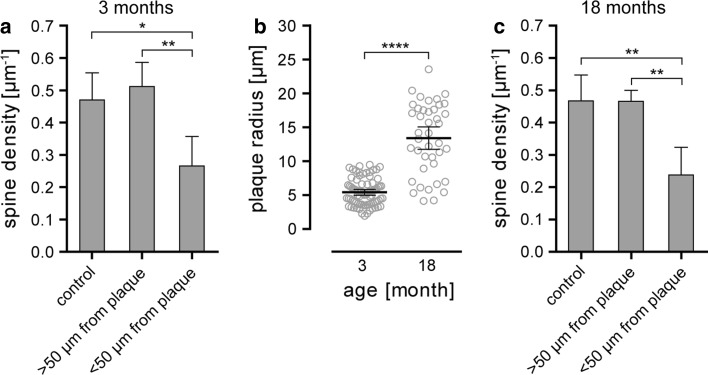
Static analysis of spine density and plaque size at 3 and 18 months of age. **a** Diagram showing the mean spine density of dendrites from control mice and transgenic mice, less and more than 50 μm away from amyloid plaques at 3 months of age. The mean spine density distant from plaques (*n* = 3 mice, 567 μm dendrite length, *n* = 278 dendritic spines) is not different from the density in control mice (*n* = 5 mice, 1,860 μm dendrite length, *n* = 869 dendritic spines), whereas the mean spine density in vicinity to plaques (*n* = 4 mice, 919 μm dendrite length, *n* = 239 dendritic spines) is significantly decreased compared to control mice (*p* < 0.05) and distant from plaques (*p* < 0.01, one-way ANOVA with Tukey–Kramer post hoc test). **b** The mean plaque radius is significantly lower at 3 months (*n* = 80) compared to 18 months (*n* = 41) of age (*p* < 0.0001, *t* test). **c** Diagram showing the mean spine density of dendrites from control mice and transgenic mice, less and more than 50 μm away from amyloid plaques at 18 months of age. The mean spine density distant from plaques (*n* = 5 mice. 1,532 μm dendrite length, *n* = 705 dendritic spines) is not different from the density in control mice (*n* = 5 mice, 1,752 μm dendrite length, *n* = 819 dendritic spines), whereas the mean spine density in vicinity to plaques (*n* = 4 mice, 611 μm dendrite length, *n* = 129 dendritic spines) is significantly decreased compared to control mice (*p* < 0.01) and distant from plaques (*p* < 0.01, one-way ANOVA with Tukey–Kramer post hoc test). *Error bars* show SD for **a**, **c**, and 95 % CI for **b**

In the present study, we used the APPPS1 mouse model, which co-expresses human amyloid precursor protein with the Swedish mutation (KM 670/671 NL) and L166P mutated presenilin 1 [46]. In these mice, the first amyloid plaques become visible before the age of 2 months and amyloid plaque load reaches almost 10 % at 8 months within the cerebral cortex [46]. These features make APPPS1 mice an excellent model to study amyloid plaque-related AD pathology. In order to investigate synapse pathology in the vicinity of plaques, these mice were intercrossed with a mouse line that expresses yellow fluorescent protein in a subset of layer III and V pyramidal neurons within the cortex (YFP-H line) [15]. Amyloid plaques were stained by the fluorescent dye methoxy-X04 [31], which crosses the blood–brain barrier and allows to study plaque growth kinetics by repeated injection before every imaging session. The implantation of an open-skull cranial window above the somatosensory cortex provides direct optical access to the brain and deep tissue imaging using two-photon microscopy can be performed over several weeks [12, 24, 55]. This experimental setup set the stage to co-investigate and correlate dendritic spine kinetics with plaque growth kinetics in two separate age groups (Fig. 1). The first group ranged from 3 to 4 months, an age when plaque growth is still very dynamic in this mouse model [22]. The second age group (18–19 months) represents the end-stage of amyloid pathology. In both groups, a single time-point analysis revealed a significant lower spine density in the vicinity (<50 μm) of amyloid plaques compared to areas distant to plaques (>50 μm) with respect to wild-type control animals (Fig. 2a, 3 months: 0.470 ± 0.084 μm^−1^ control, 0.511 ± 0.075 μm^−1^ > 50 μm from plaque, 0.264 ± 0.092 μm^−1^ < 50 μm from plaque, *p* < 0.05; Fig. 2c, 18 months: 0.467 ± 0.081 μm^−1^ control, 0.466 ± 0.034 μm^−1^ > 50 μm from plaque, 0.238 ± 0.086 μm^−1^ < 50 μm from plaque, *p* < 0.01, one-way ANOVA with Tukey–Kramer post hoc test, errors represent SD). The dendritic spine density in the vicinity of amyloid plaques did not significantly differ between age groups (Supplementary Fig. a). It is important to mention that we were actively looking for spine-bearing dendrites to analyze in the surrounding volume of amyloid plaques, which were subjectively more difficult to find, yet still present, in the older cohort. During the same time interval, plaque size increased by almost threefold, from a mean radius of 5.413 μm at 3 months to 13.410 μm at 18 months (Fig. 2b, 95 % CI 4.980–5.846 μm and 11.750–15.060 μm, *p* < 0.0001, *t* test).

**Fig. 3:**
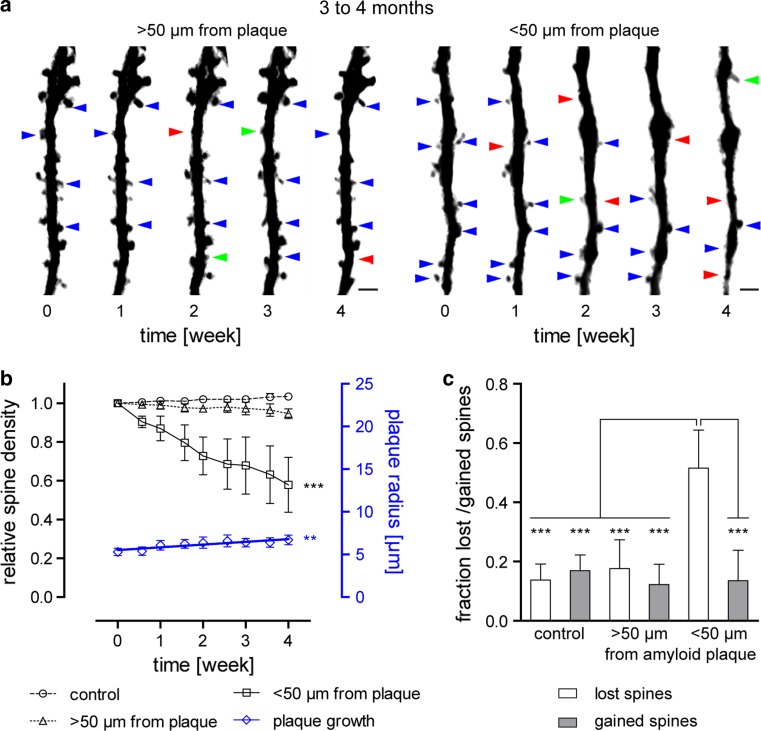
Kinetics of dendritic spines and amyloid plaque size from 3 to 4 months of age. **a** Representative time series of YFP-labeled dendrites and spines shown as maximum intensity projections, more and less than 50 μm distant from plaques. *Blue arrows* indicate maintained spines, *red arrows* lost spines, and *green arrows* gained spines (only some spines are exemplarily marked). *Scale bar* represents 2 μm. **b** Relative spine densities (density normalized to time point 0) are presented by *black symbols*. Data from control mice (*n* = 5) are shown as *circles*, > 50 μm away from plaque (*n* = 3) are indicated by *triangles* and <50 μm away from plaques (*n* = 4) as *squares*. *Error bars* show SEM. The decline in spine density in vicinity to plaques is significant (*p* < 0.001, repeated measures ANOVA with Tukey–Kramer post hoc test). Plaque radius is indicated by *blue diamonds*. Linear regression revealed a significant increase in size over 4 weeks (*n* = 80 plaques from seven mice, slope 0.320 ± 0.066 μm week^−1^, *p* < 0.01, *F* test, *F* = 23.811, DFn = 1, DFd = 7). *Error bars* indicate 95 % CI. **c** Diagram of the fraction of lost and gained spines over 4 weeks. Spine elimination is significantly increased for dendrites <50 μm distant to plaques compared to dendrites from control animals (*p* < 0.001, one-way ANOVA with Tukey–Kramer post hoc test). Spine formation remained constant under all conditions. *Error bars* indicate SD

**Fig. 4:**
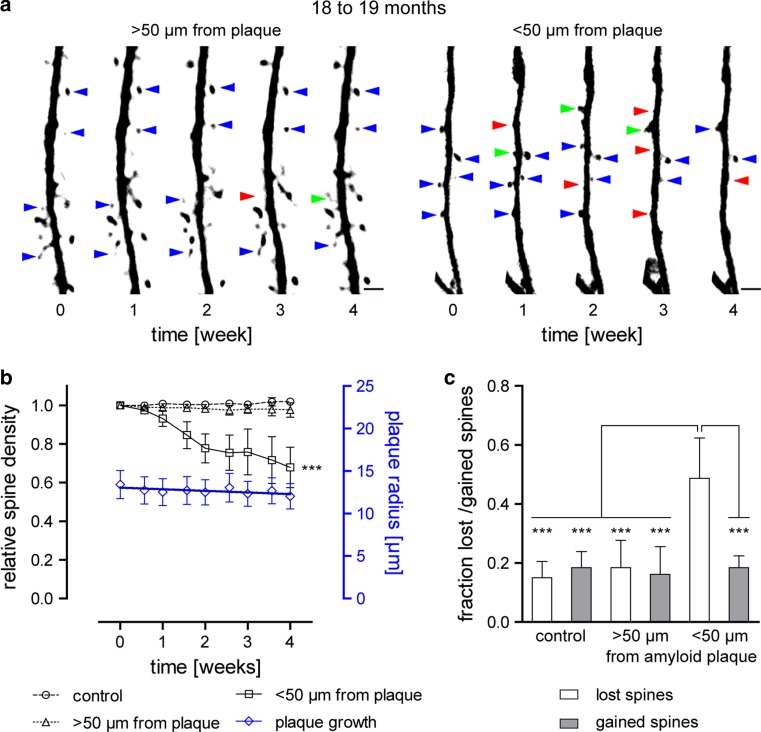
Kinetics of dendritic spines and amyloid plaque size from 18 to 19 months of age. **a** Representative time series of YFP-labeled dendrites and spines shown as maximum intensity projections, more and less than 50 μm distant from plaques. *Blue arrows* indicate maintained spines, *red arrows* lost spines, and *green arrows* gained spines (only some spines are exemplarily marked). *Scale bar* represents 2 μm. **b** Relative spine densities (density normalized to time point 0) are presented by *black symbols*. Data from control mice (*n* = 5) are shown as *circles*, >50 μm away from plaque (*n* = 5) are indicated by *triangles*, and <50 μm away from plaques (*n* = 4) as *squares*. *Error bars* show SEM. The decline in spine density in vicinity to plaques is significant (*p* < 0.001, repeated measures ANOVA with Tukey–Kramer post hoc test). Plaque radius is indicated by *blue diamonds*. Linear regression revealed a slight decrease in size over 4 weeks, which is not significant (*n* = 41 plaques from eight mice, slope −0.189 ± 0.084 μm week^−1^, *p* > 0.05, *F* test, *F* = 4.992, DFn = 1, DFd = 7). *Error bars* indicate 95 % CI. **c** Diagram of the fraction of lost and gained spines over 4 weeks. Spine elimination is significantly increased for dendrites <50 μm distant to plaques compared to dendrites from control animals (*p* < 0.001, one-way ANOVA with Tukey–Kramer post hoc test). Spine formation remained constant under all conditions. *Error bars* indicate SD

After this single time-point analysis, we focused on the kinetics of amyloid plaque growth and spine loss over 4 weeks. Therefore, the spine density at mature plaques and at nascent plaques was analyzed in young and old cohorts. Within the peri-plaque region (<50 μm around plaques) we found a significant reduction in spine density in both young and aged mice (Fig. 3a, b 57.9 ± 28.3 % at 4 months and Fig. 4a, b 67.8 ± 20.8 % at 19 months, *p* < 0.001, repeated measures ANOVA with Tukey–Kramer post hoc test). Over the same time period, no spine loss was detected >50 μm distant from plaques or in wild-type animals having no plaques at all. Notably, spine loss kinetics were not significantly different between both age groups (Supplementary Fig. c). Long-term in vivo imaging makes it possible to follow individual spines over time and to determine the fraction of lost and gained spines. Consequently, we could identify the factor responsible for a net decrease in spine density. Such an analysis revealed that the overall decline in spine density could be attributed to a relative increase in the fraction of lost spines over 4 weeks. The fraction of gained spines, on the other hand, remained the same when compared to areas distant to plaques or in control animals in both age groups (Figs. 3c, 4c, *p* < 0.001, one-way ANOVA with Tukey–Kramer post hoc test). In other words, the observed loss of spines at pre-existing plaques is caused by an increase in spine elimination rather than by a malfunction in the process to form new spines.

**Fig. 5:**
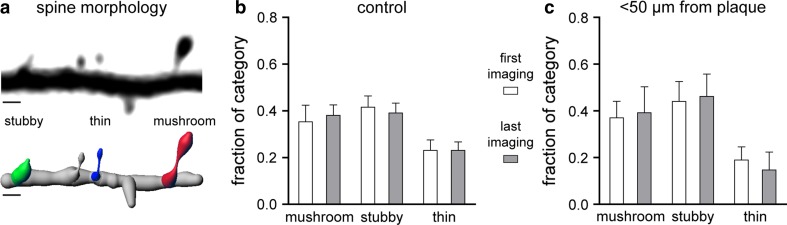
Morphology of dendritic spines from 18 to 19 months of age. **a** Image showing a YFP labeled dendrite (*black*) and a 3D reconstruction of the same dendrite below. Reconstruction was done for all spines and for each category of spine morphology; examples are shown (“thin” = *blue*, “stubby” = *green*, “mushroom” = *red*). *Scale bar* represents 1 μm. The *graphs* show the fractions of spine morphology categories in control mice **b** (*n* = 5 mice, 842 μm dendrite length, *n* = 361 dendritic spines) and for dendrites <50 μm away from plaques **c** (*n* = 4 mice, 611 μm dendrite length, *n* = 129 dendritic spines). There was no change in the fraction of any category over time. A comparison between the control group and dendrites <50 μm away from plaques within each category also did not reveal any statistically significant differences (Chi-squared test). *Error bars* indicate 95 % CI

We further investigated whether a specific morphological subtype of dendritic spine is lost in the vicinity of amyloid plaques. With this aim, we classified dendritic spines into “thin”, “stubby”, and “mushroom” spines based on their morphology [21] (Fig. 5a). For every dendrite we analyzed the first and last imaging time-point, 4 weeks later. We expected to see differences, if any, in the old cohort at 18–19 months of age, since pathology here is in an advanced stage. However, we found no significant differences in each morphological category over time (Fig. 5b, c, Chi-square test). There were also no differences in the distribution of the three dendritic spine subtypes between the control and in the vicinity of plaques (Fig. 5b, c, Chi-square test). Thus, we conclude that no specific morphological subtype is preferentially eliminated.

Plaque growth was quantified by converting the cubic root from volumes in 3D images to yield a linear measure, analogous to the radius [22]. In the young cohort, we could confirm a significant growth of 0.320 ± 0.066 μm week^−1^, which had previously been reported by Hefendehl et al. [22] (Fig. 3b, *p* < 0.01, *F* test, *F* = 23.811, DFn = 1, DFd = 7). In contrast, plaque radius in aged mice remained unchanged (Fig. 4b, −0.189 ± 0.084 μm week^−1^). Lack of plaque growth is supported by a similar finding in aged Tg2576 mice, another mouse model of AD [8, 9].

**Fig. 6:**
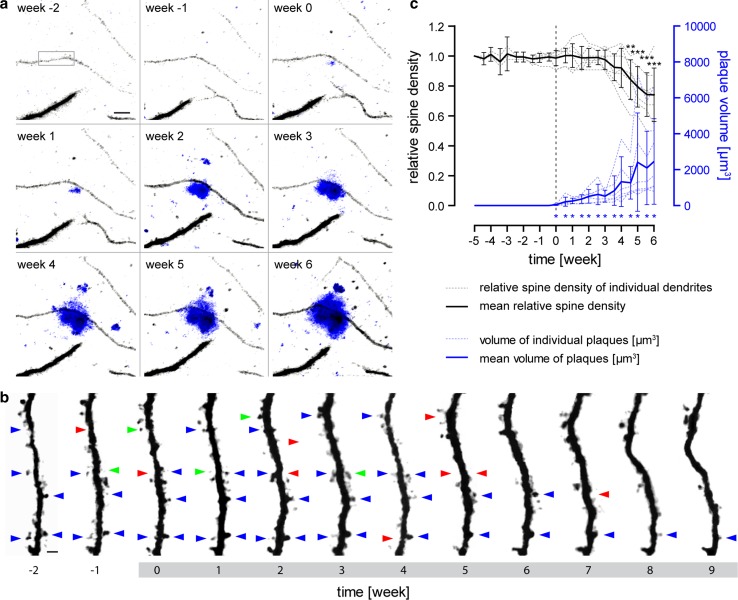
Spine density kinetics before and during the formation of amyloid plaques. **a** Maximum intensity projections of two-photon in vivo images of YFP-labeled dendrites (*grey*) are shown in a weekly imaging interval. At week 0, a new plaque (*blue*) appeared in direct vicinity to the dendrite in the *center*. The *black rectangle* marks the dendritic segment, which is shown in greater magnification in **b**. Important to note, the dendrite does not take course directly through the plaque, but the plaque is located above the dendrite (for a 3D view see supplementary figure). *Scale bar* indicates 10 μm. **b** Time series of the maximum intensity projected YFP labeled dendrite from **a**. The *grey* highlighted time scale indicates the time period when the amyloid plaque is already present. *Blue arrows* indicate maintained spines, *red arrows* lost spines, and *green arrows* gained spines (only some spines are exemplarily marked). *Scale bar* represents 2 μm. **c** Quantification of the dendritic spine kinetics are shown in *black* and plaque growth kinetics in *blue*. Spine densities were normalized to the spine densities at the first time point. The time point when amyloid appeared was set to 0 and is marked by a *dashed line*. Individual traces from seven dendrites (*n* = 2 mice, 444 µm dendrite length, *n* = 247 dendritic spines) are indicated by *dashed lines*, whereas the *solid line* shows mean with 95 % CI. Dendritic spine loss became first significant 4 weeks after plaque appearance (*p* < 0.01, repeated measures ANOVA with Tukey–Kramer post hoc test). In contrast, the increase in amyloid plaque volume became significant directly with appearance (*p* < 0.05, Wilcoxon signed-rank test against theoretical value 0 μm^3^)

Finally, we analyzed the kinetics of individual dendritic spines before and during the de novo formation of new plaques. The observation of plaque birth is a very rare event [8, 22, 41, 61] and for spine analysis to be feasible in proximity to an appearing plaque, two prerequisites have to be fulfilled. Firstly, plaque birth has to occur in the vicinity of a dendrite lying mainly in the imaging plane (due to resolution limits in the axial direction) [12, 55]. Secondly, the resolution of the images has to be high enough to resolve individual dendritic spines over long periods of time. To meet both criteria, 12 volumes of the dimensions 283 × 283 × 300 μm^3^ (totaling 0.288 mm^3^) were imaged over a period of 3 months. In these large volumes of high resolution, we were able to detect the exceptional event of plaque birth in close proximity to dendrites seven times over. Imaging was started at 2–3 months of age when few amyloid plaques were present, with new plaques expected to form during the following months [22]. To the best of our knowledge, this is the first time that individual dendritic spines were monitored before and during the formation of an amyloid plaque in their proximity (Fig. 6a, b). Interestingly, the spine density of these dendrites remained unchanged prior to plaque formation and did not decline immediately after the plaques first appeared (Fig. 6c, total length of dendritic segments 444 μm, *n* = 247 dendritic spines). A significant reduction in spine density did however occur 4.5 weeks after initial amyloid plaque formation (*p* < 0.01, repeated measures one-way ANOVA with Tukey–Kramer post hoc test). Over the same time period, plaque volume increased significantly immediately after the plaque’s appearance (Fig. 6c, *p* < 0.05, Wilcoxon signed-rank test against theoretical value 0 μm^3^). Thus, there seems to be a latency of about 4 weeks between plaque formation and the onset of dendritic spine elimination in the vicinity of plaques.

## Discussion

We co-investigated and correlated amyloid plaque growth with dendritic spine kinetics in APPPS1 mice by long-term two-photon in vivo imaging. Several studies previously reported dendritic spine loss around amyloid plaques in a range of different mouse models and brain regions by applying both ex vivo and in vivo approaches [6, 19, 32, 34, 40, 53, 54, 59]. Here, we were able to confirm spine loss in proximity of plaques in the APPPS1 mouse model. However, the primary focus of this study was to analyze the underlying kinetics of spine loss around plaques. We were especially interested in the chronology: Which event comes first, dendritic spine loss or plaque formation? While a recent study investigated changes of neuritic curvature after plaque appearance [41], we were able to observe the fate of individual dendritic spines not only after the appearance of fibrillar plaques, but also before. We investigated spine loss kinetics in two age groups: a young cohort, 3–4 months old, when plaque growth is highly dynamic and an older cohort, 18–19 months old, when the plaque pathology is in the terminal stages.

As expected, we noted significant plaque growth in the young cohort, in line with several other studies [8, 10, 22, 61]. Moreover, our analysis showed a similar rate of plaque growth (0.320 ± 0.066 μm week^−1^, Fig. 3b) as reported by Hefendehl et al. [22] in the same mouse model and at a similar age (~0.3 μm week^−1^). Hefendehl et al. imaged mice up to an age of 10 months and observed no decline in plaque growth kinetics by that age. However, when we extended the observation period to an age of 18–19 months, we found that plaque growth declined essentially to zero (Fig. 4b). This finding corroborates the results from two other studies which also found no plaque growth in aged Tg2576 mice [8, 9]. Further support for a slow and gradual growth of plaques over extended periods of time is given by the large difference in the mean plaque sizes between the two age cohorts (Fig. 2b).

In the second part of our study, we identified a significant loss of spines in close proximity to amyloid plaques (<50 μm) compared to areas farther away from plaques and in age-matched control animals. Interestingly, similar results were found for both age cohorts. Thus, when monitoring the relative spine density over 4 weeks at already-existing plaques, we found a reduction to 57.9 ± 28.3 % in young mice and 67.8 ± 20.8 % in aged mice (Figs. 3a, b, 4a, b). Linear regression analysis showed no statistically significant difference between these results (Suppl. Fig. c). This finding suggests that spine loss around plaques persists for more than 1 year in this mouse model. Taking also into consideration the results from plaque growth kinetics, we can envision a situation in which plaques are steadily growing and, of course, the volume around the plaques, where spine density is affected, increases as well. Nevertheless, even when plaque growth is not detectable anymore in aged mice, synapses are still being lost. In this context, it is worth noting that the spine density of dendrites in the surrounding volume of amyloid plaques in both age groups were quite similar (Suppl. Fig. a), while the number of healthy-looking dendrites was subjectively decreased in older mice. A previous study made a similar observation in aged (18–24 months) Tg2576 mice. Over 40 % aspiny dendrites were found in the surrounding area of plaques compared to about 15 % in control animals [53].

In the adult rodent brain, a small fraction of dendritic spines undergoes a certain turnover with spines being newly gained or lost, while the majority of spines is stable over long periods of time [20, 25, 58, 63]. In Tg2576 mice, increased spine elimination was found in proximity to plaques over 1 h [53]. In another AD mouse model (PSAPP), an increase in both spine formation as well as elimination was reported over 4–5 weeks [59].

Thus, besides confirming the fact that spines are lost around amyloid plaques, we also determined whether the spine loss was attributable to a decrease in gained spines or an increase in lost spines. In fact, we did not observe any changes in the fractions of gained spines, but a strong and significant increase in the fraction of lost spines measured in the vicinity of plaques over 4 weeks (Figs. 3c, 4c). In parallel to the reduced spine density, the fractions of lost spines were equally elevated in both age groups (Suppl. Fig. b). Moreover, the increased number of lost spines was due to the elimination of stable spines (data not shown). A further analysis revealed no preferential decline of a specific morphological subtype of dendritic spines over time within the old cohort (Fig. 5a–c). There was also no difference when comparing the morphological classification of dendritic spines from control animals with dendrites from the region around amyloid plaques (Fig. 5b, c). In conclusion, we detected a disturbed spine turnover within the peri-plaque region that was caused by a loss of stable spines. These stable spines are considered to represent the structural basis for long-term information storage [2, 4]. Increased spine elimination might be related to a higher concentration of toxic amyloid-β species in proximity to amyloid plaques as previously proposed [27, 30, 34, 49].

However, intriguingly, spine density did not decline immediately after the first appearance of a plaque, but rather following a delay of at least 4 weeks. This finding answers the question of what comes first, spine loss or amyloid plaque formation, [52] in favor of the latter. There even seems to be a significant delay between amyloid plaque formation and commencement of dendritic spine loss. Combining this finding with the result that spine loss around plaques is a protracted process taking place over a long period of time, the present study may explain the delay of 5–10 years between the accumulation of amyloid-β in human brains and onset of cognitive decline [50, 51]. However, we would like to emphasize that dendritic spine loss is only one part of the underlying multifactorial pathology of AD that contributes to cognitive decline besides neuron death, tauopathy, and hyperexcitability, to mention just some. Notwithstanding, the findings presented here may give hope to the possibility that even after initial plaque formation there might be a therapeutic window where potentially irreversible structural damage to synapses could still be prevented.

## Electronic supplementary material

Below is the link to the electronic supplementary material.

Supplementary material 1 (TIFF 1762 kb) Direct comparison of spine densities (**a**), fraction of lost spines (**b**), and spine density kinetics (**c**) at 3 and 18 months of age for dendrites less than 50 µm away from amyloid plaques.* Error bars* indicate SD for a and b and SEM for c. **d** 3D volume rendered view from the two-photon in vivo image from figure 5a. YFP-labeled dendrites are shown in* green* and methoxy-X04 stained amyloid plaques in* blue*. The xz- and yz-view highlights that the investigated dendrite from figure 5a does not directly pass through the plaque. The* black rectangle* marks the picture detail from figure 5a and the scale represent 10 µm

## References

[1] Alpár A, Ueberham U, Brückner MK, Seeger G, Arendt T, Gärtner U (2006). Different dendrite and dendritic spine alterations in basal and apical arbors in mutant human amyloid precursor protein transgenic mice. Brain Res.

[2] Alvarez VA, Sabatini BL (2007). Anatomical and physiological plasticity of dendritic spines. Annu Rev Neurosci.

[3] Arendt T (2009). Synaptic degeneration in Alzheimer’s disease. Acta Neuropathol.

[4] Bhatt DH, Zhang S, Gan W-B (2009). Dendritic spine dynamics. Annu Rev Physiol.

[5] Bittner T, Fuhrmann M, Burgold S, Jung CKE, Volbracht C, Steiner H, Mitteregger G, Kretzschmar HA, Haass C, Herms J (2009). Gamma-secretase inhibition reduces spine density in vivo via an amyloid precursor protein-dependent pathway. J Neurosci Off J Soc Neurosci.

[6] Bittner T, Fuhrmann M, Burgold S, Ochs SM, Hoffmann N, Mitteregger G, Kretzschmar H, LaFerla FM, Herms J (2010). Multiple events lead to dendritic spine loss in triple transgenic Alzheimer’s disease mice. PLoS ONE.

[7] Bolmont T, Haiss F, Eicke D, Radde R, Mathis CA, Klunk WE, Kohsaka S, Jucker M, Calhoun ME (2008). Dynamics of the microglial/amyloid interaction indicate a role in plaque maintenance. J Neurosci Off J Soc Neurosci.

[8] Burgold S, Bittner T, Dorostkar MM, Kieser D, Fuhrmann M, Mitteregger G, Kretzschmar H, Schmidt B, Herms J (2011). In vivo multiphoton imaging reveals gradual growth of newborn amyloid plaques over weeks. Acta Neuropathol.

[9] Christie RH, Bacskai BJ, Zipfel WR, Williams RM, Kajdasz ST, Webb WW, Hyman BT (2001). Growth arrest of individual senile plaques in a model of Alzheimer’s disease observed by in vivo multiphoton microscopy. J Neurosci Off J Soc Neurosci.

[10] Condello C, Schain A, Grutzendler J (2011). Multicolor time-stamp reveals the dynamics and toxicity of amyloid deposition. Scientific Rep.

[11] DeKosky ST, Scheff SW (1990). Synapse loss in frontal cortex biopsies in Alzheimer’s disease: correlation with cognitive severity. Ann Neurol.

[12] Denk W, Strickler JH, Webb WW (1990) Two-photon laser scanning fluorescence microscopy. Science (New York) 248:73–76 10.1126/science.2321027 2321027

[13] Dickson DW (1997). The pathogenesis of senile plaques. J Neuropathol Exp Neurol.

[14] Duyckaerts C, Potier M-C, Delatour B (2008). Alzheimer disease models and human neuropathology: similarities and differences. Acta Neuropathol.

[15] Feng G, Mellor RH, Bernstein M, Keller-Peck C, Nguyen QT, Wallace M, Nerbonne JM, Lichtman JW, Sanes JR (2000). Imaging neuronal subsets in transgenic mice expressing multiple spectral variants of GFP. Neuron.

[16] Fiala JC, Spacek J, Harris KM (2002). Dendritic spine pathology: cause or consequence of neurological disorders?. Brain Res Brain Res Rev.

[17] Fuhrmann M, Bittner T, Jung CKE, Burgold S, Page RM, Mitteregger G, Haass C, LaFerla FM, Kretzschmar H, Herms J (2010). Microglial Cx3cr1 knockout prevents neuron loss in a mouse model of Alzheimer’s disease. Nat Neurosci.

[18] Fuhrmann M, Mitteregger G, Kretzschmar H, Herms J (2007). Dendritic pathology in prion disease starts at the synaptic spine. J Neurosci Off J Soc Neurosci.

[19] Grutzendler J, Helmin K, Tsai J, Gan W-B (2007). Various dendritic abnormalities are associated with fibrillar amyloid deposits in Alzheimer’s disease. Ann N Y Acad Sci.

[20] Grutzendler J, Kasthuri N, Gan WB (2002). Long-term dendritic spine stability in the adult cortex. Nature.

[21] Harris KM, Jensen FE, Tsao B (1992). Three-dimensional structure of dendritic spines and synapses in rat hippocampus (CA1) at postnatal day 15 and adult ages: implications for the maturation of synaptic physiology and long-term potentiation. J Neurosci.

[22] Hefendehl JK, Wegenast-Braun BM, Liebig C, Eicke D, Milford D, Calhoun ME, Kohsaka S, Eichner M, Jucker M (2011). Long-term in vivo imaging of β-amyloid plaque appearance and growth in a mouse model of cerebral β-amyloidosis. J Neurosci Off J Soc Neurosci.

[23] Heinonen O, Soininen H, Sorvari H, Kosunen O, Paljärvi L, Koivisto E, Riekkinen PJ (1995). Loss of synaptophysin-like immunoreactivity in the hippocampal formation is an early phenomenon in Alzheimer’s disease. Neuroscience.

[24] Helmchen F, Denk W (2005). Deep tissue two-photon microscopy. Nat Methods.

[25] Holtmaat AJGD, Trachtenberg JT, Wilbrecht L, Shepherd GM, Zhang X, Knott GW, Svoboda K (2005). Transient and persistent dendritic spines in the neocortex in vivo. Neuron.

[26] Honer WG, Dickson DW, Gleeson J, Davies P (1992). Regional synaptic pathology in Alzheimer’s disease. Neurobiol Aging.

[27] Hsieh H, Boehm J, Sato C, Iwatsubo T, Tomita T, Sisodia S, Malinow R (2006). AMPAR removal underlies Abeta-induced synaptic depression and dendritic spine loss. Neuron.

[28] Jacobsen JS, Wu C–C, Redwine JM, Comery TA, Arias R, Bowlby M, Martone R, Morrison JH, Pangalos MN, Reinhart PH, Bloom FE (2006). Early-onset behavioral and synaptic deficits in a mouse model of Alzheimer’s disease. Proc Natl Acad Sci USA.

[29] Jung CKE, Fuhrmann M, Honarnejad K, Van Leuven F, Herms J (2011). Role of presenilin 1 in structural plasticity of cortical dendritic spines in vivo. J Neurochem.

[30] Kamenetz F, Tomita T, Hsieh H, Seabrook G, Borchelt D, Iwatsubo T, Sisodia S, Malinow R (2003). APP processing and synaptic function. Neuron.

[31] Klunk WE, Bacskai BJ, Mathis CA, Kajdasz ST, McLellan ME, Frosch MP, Debnath ML, Holt DP, Wang Y, Hyman BT (2002). Imaging Abeta plaques in living transgenic mice with multiphoton microscopy and methoxy-X04, a systemically administered Congo red derivative. J Neuropathol Exp Neurol.

[32] Knafo S, Alonso-Nanclares L, Gonzalez-Soriano J, Merino-Serrais P, Fernaud-Espinosa I, Ferrer I, DeFelipe J (2009) Widespread changes in dendritic spines in a model of Alzheimer’s disease. Cereb Cortex (New York) 19:586–592. doi:10.1093/cercor/bhn111 10.1093/cercor/bhn111 18632740

[33] Knobloch M, Mansuy IM (2008). Dendritic spine loss and synaptic alterations in Alzheimer’s disease. Mol Neurobiol.

[34] Koffie RM, Meyer-Luehmann M, Hashimoto T, Adams KW, Mielke ML, Garcia-Alloza M, Micheva KD, Smith SJ, Kim ML, Lee VM, Hyman BT, Spires-Jones TL (2009). Oligomeric amyloid beta associates with postsynaptic densities and correlates with excitatory synapse loss near senile plaques. Proc Natl Acad Sci USA.

[35] Lanz TA, Carter DB, Merchant KM (2003). Dendritic spine loss in the hippocampus of young PDAPP and Tg2576 mice and its prevention by the ApoE2 genotype. Neurobiol Dis.

[36] Lassmann H, Weiler R, Fischer P, Bancher C, Jellinger K, Floor E, Danielczyk W, Seitelberger F, Winkler H (1992). Synaptic pathology in Alzheimer’s disease: immunological data for markers of synaptic and large dense-core vesicles. Neuroscience.

[37] Liebscher S, Meyer-Luehmann M (2012) A Peephole into the brain: neuropathological features of Alzheimer’s disease revealed by in vivo two-photon imaging. Frontiers Psychiatry/Frontiers Res Found 3:26. doi:10.3389/fpsyt.2012.00026 10.3389/fpsyt.2012.00026 PMC3317174 22485096

[38] Lue LF, Brachova L, Civin WH, Rogers J (1996). Inflammation, A beta deposition, and neurofibrillary tangle formation as correlates of Alzheimer’s disease neurodegeneration. J Neuropathol Exp Neurol.

[39] Masliah E, Terry RD, DeTeresa RM, Hansen LA (1989). Immunohistochemical quantification of the synapse-related protein synaptophysin in Alzheimer disease. Neurosci Lett.

[40] Merino-Serrais P, Knafo S, Alonso-Nanclares L, Fernaud-Espinosa I, DeFelipe J (2011). Layer-specific alterations to CA1 dendritic spines in a mouse model of Alzheimer’s disease. Hippocampus.

[41] Meyer-Luehmann M, Spires-Jones TL, Prada C, Garcia-Alloza M, de Calignon A, Rozkalne A, Koenigsknecht-Talboo J, Holtzman DM, Bacskai BJ, Hyman BT (2008). Rapid appearance and local toxicity of amyloid-beta plaques in a mouse model of Alzheimer’s disease. Nature.

[42] Nelson PT, Alafuzoff I, Bigio EH, Bouras C, Braak H, Cairns NJ, Castellani RJ, Crain BJ, Davies P, Del Tredici K, Duyckaerts C, Frosch MP, Haroutunian V, Hof PR, Hulette CM, Hyman BT, Iwatsubo T, Jellinger KA, Jicha GA, Kövari E, Kukull WA, Leverenz JB, Love S, Mackenzie IR, Mann DM, Masliah E, McKee AC, Montine TJ, Morris JC, Schneider JA, Sonnen JA, Thal DR, Trojanowski JQ, Troncoso JC, Wisniewski T, Woltjer RL, Beach TG (2012). Correlation of Alzheimer disease neuropathologic changes with cognitive status: a review of the literature. J Neuropathol Exp Neurol.

[43] Nimchinsky EA, Sabatini BL, Svoboda K (2002). Structure and function of dendritic spines. Annu Rev Physiol.

[44] Penzes P, Cahill ME, Jones KA, VanLeeuwen J-E, Woolfrey KM (2011). Dendritic spine pathology in neuropsychiatric disorders. Nat Neurosci.

[45] Perez-Cruz C, Nolte MW, van Gaalen MM, Rustay NR, Termont A, Tanghe A, Kirchhoff F, Ebert U (2011). Reduced spine density in specific regions of CA1 pyramidal neurons in two transgenic mouse models of Alzheimer’s disease. J Neurosci Off J Soc Neurosci.

[46] Radde R, Bolmont T, Kaeser SA, Coomaraswamy J, Lindau D, Stoltze L, Calhoun ME, Jäggi F, Wolburg H, Gengler S, Haass C, Ghetti B, Czech C, Hölscher C, Mathews PM, Jucker M (2006). Abeta42-driven cerebral amyloidosis in transgenic mice reveals early and robust pathology. EMBO Rep.

[47] Scheff SW, DeKosky ST, Price DA (1990). Quantitative assessment of cortical synaptic density in Alzheimer’s disease. Neurobiol Aging.

[48] Scheff SW, Price DA, Schmitt FA, DeKosky ST, Mufson EJ (2007). Synaptic alterations in CA1 in mild Alzheimer disease and mild cognitive impairment. Neurology.

[49] Shankar GM, Li S, Mehta TH, Garcia-Munoz A, Shepardson NE, Smith I, Brett FM, Farrell MA, Rowan MJ, Lemere CA, Regan CM, Walsh DM, Sabatini BL, Selkoe DJ (2008). Amyloid-beta protein dimers isolated directly from Alzheimer’s brains impair synaptic plasticity and memory. Nat Med.

[50] Sperling RA, Aisen PS, Beckett LA, Bennett DA, Craft S, Fagan AM, Iwatsubo T, Jack CR, Kaye J, Montine TJ, Park DC, Reiman EM, Rowe CC, Siemers E, Stern Y, Yaffe K, Carrillo MC, Thies B, Morrison-Bogorad M, Wagster MV, Phelps CH (2011). Toward defining the preclinical stages of Alzheimer’s disease: recommendations from the National Institute on Aging-Alzheimer’s Association workgroups on diagnostic guidelines for Alzheimer’s disease. Alzheimer Dementia J Alzheimer’s Assoc.

[51] Sperling RA, Laviolette PS, O’Keefe K, O’Brien J, Rentz DM, Pihlajamaki M, Marshall G, Hyman BT, Selkoe DJ, Hedden T, Buckner RL, Becker JA, KA Johnson (2009). Amyloid deposition is associated with impaired default network function in older persons without dementia. Neuron.

[52] Spires-Jones T, Knafo S (2012). Spines, plasticity, and cognition in Alzheimer’s model mice. Neural Plasticity.

[53] Spires-Jones TL, Meyer-Luehmann M, Osetek JD, Jones PB, Stern EA, Bacskai BJ, Hyman BT (2007). Impaired spine stability underlies plaque-related spine loss in an Alzheimer’s disease mouse model. Am J Pathol.

[54] Spires TL, Meyer-Luehmann M, Stern EA, McLean PJ, Skoch J, Nguyen PT, Bacskai BJ, Hyman BT (2005). Dendritic spine abnormalities in amyloid precursor protein transgenic mice demonstrated by gene transfer and intravital multiphoton microscopy. J Neurosci Off J Soc Neurosci.

[55] Svoboda K, Yasuda R (2006). Principles of two-photon excitation microscopy and its applications to neuroscience. Neuron.

[56] Terry RD, Masliah E, Salmon DP, Butters N, DeTeresa R, Hill R, Hansen LA, Katzman R (1991). Physical basis of cognitive alterations in Alzheimer’s disease: synapse loss is the major correlate of cognitive impairment. Ann Neurol.

[57] Thal DR, Capetillo-Zarate E, Del Tredici K, Braak H (2006) The development of amyloid beta protein deposits in the aged brain. Sci Aging Knowl Environ (SAGE KE) 2006:re1. doi:10.1126/sageke.2006.6.re1 10.1126/sageke.2006.6.re1 16525193

[58] Trachtenberg JT, Chen BE, Knott GW, Feng G, Sanes JR, Welker E, Svoboda K (2002). Long-term in vivo imaging of experience-dependent synaptic plasticity in adult cortex. Nature.

[59] Tsai J, Grutzendler J, Duff K, Gan W-B (2004). Fibrillar amyloid deposition leads to local synaptic abnormalities and breakage of neuronal branches. Nat Neurosci.

[60] Walsh DM, Selkoe DJ (2004). Deciphering the molecular basis of memory failure in Alzheimer’s disease. Neuron.

[61] Yan P, Bero AW, Cirrito JR, Xiao Q, Hu X, Wang Y, Gonzales E, Holtzman DM, Lee J-M (2009). Characterizing the appearance and growth of amyloid plaques in APP/PS1 mice. J Neurosc Off J Soc Neurosci.

[62] Yuste R, Majewska A (2001). On the function of dendritic spines. Neuroscientist Rev J Bringing Neurobiol Neurol Psychiatry.

[63] Zuo Y, Lin A, Chang P, Gan W-B (2005). Development of long-term dendritic spine stability in diverse regions of cerebral cortex. Neuron.

