# Supplementary figures and images for: Amyloid plaque formation precedes dendritic spine loss
Source: Acta Neuropathol. 2012 Sep 21;124(6):797–807. doi: 10.1007/s00401-012-1047-8 (PMC3508278; doi:10.1007/s00401-012-1047-8)

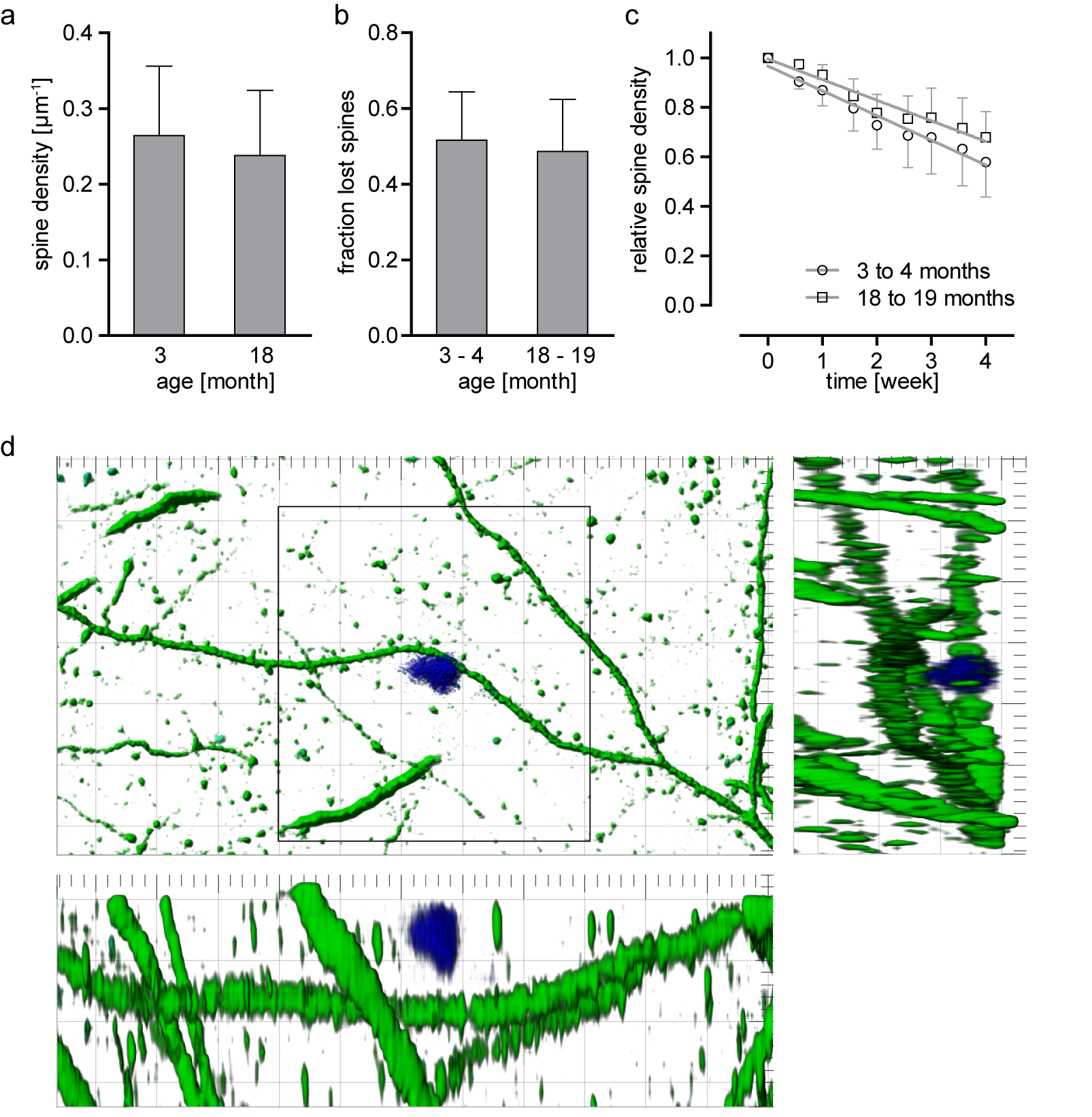

Supplement: Supplementary file 1 — Supplementary material 1 (TIFF 1762 kb) Direct comparison of spine densities (a), fraction of lost spines (b), and spine density kinetics (c) at 3 and 18 months of age for dendrites less than 50 µm away from amyloid plaques. Error bars indicate SD for a and b and SEM for c. d 3D volume rendered view from the two-photon in vivo image from figure 5a. YFP-labeled dendrites are shown in green and methoxy-X04 stained amyloid plaques in blue. The xz- and yz-view highlights that the investigated dendrite from figure 5a does not directly pass through the plaque. The black rectangle marks the picture detail from figure 5a and the scale represent 10 µm [file 401_2012_1047_MOESM1_ESM.tif]
